# Carcinoembryonic antigen levels correlated with advanced disease in medullary thyroid cancer

**DOI:** 10.1186/s40463-018-0303-x

**Published:** 2018-09-17

**Authors:** Sena Turkdogan, Véronique-Isabelle Forest, Michael P. Hier, Michael Tamilia, Anca Florea, Richard J. Payne

**Affiliations:** 1 McGill University Health Center, Montreal, QC Canada; 2 Department of Otolaryngology-Head and Neck Surgery, Sir Mortimer B. Davis-Jewish General Hospital, Montreal, Canada; 3 Department of Endocrinology and Metabolism, Sir Mortimer B. Davis-Jewish General Hospital, Montreal, Canada; 4 Department of Pathology, Sir Mortimer B. Davis-Jewish General Hospital, Montreal, Canada; 5 Department of Otolaryngology-Head and Neck Surgery, McGill University Health Centre, Montreal, Canada

**Keywords:** Medullary thyroid carcinoma, Carcinoembryonic antigen, Tumor marker, Advanced disease, Mortality, Calcitonin

## Abstract

**Background:**

Medullary thyroid cancer (MTC) cells are capable of secreting various tumor markers including calcitonin and carcinoembyronic antigen (CEA). The purpose of this study is to determine whether abnormal CEA levels may be used as a tumor marker to predict the severity of disease in MTC.

**Methods:**

A retrospective analysis was completed for 33 patients with MTC who had preoperative serum CEA levels. Univariate and multivariate analyses were used to quantify the relationship between serum CEA levels and tumor stage and prognosis.

**Results:**

On multivariate analysis, elevated preoperative CEA levels were significantly associated with the size and stage of tumor, distant metastasis, decreased biochemical cure, and mortality. There was a significant association between tumor size greater than 37 mm and elevated CEA levels (> 271 ng/ml). There was also a positive correlation with increased cancer stage (> 377 ng/ml), distant metastasis (> 405 ng/ml), and contralateral compartment location of lymph node metastasis (> 162 ng/ml). When pre-operative CEA levels are > 500 ng/ml, patient mortality was 67%.

**Conclusion:**

In this study, both pre-operative calcitonin and CEA levels were significantly correlated with the extent of disease in MTC. While calcitonin has a linear relationship with disease progression, abnormal CEA levels were a better indicator of advanced disease. CEA levels > 271 ng/ml are significant for advanced tumor size and staging, metastasis to the central compartment, and decreased chance of biochemical cure. CEA levels greater than 500 ng/ml are associated with significant patient mortality.

## Background

Medullary thyroid cancer (MTC) is a neuroendocrine tumor of the parafollicular or C cells of the thyroid gland, and currently accounts for approximately 5 to 10% of all thyroid cancers [1, 2]. The clinical course of MTC can vary from an extremely indolent tumor that remains unchanged for years to an aggressive variant associated with a high mortality rate. The majority of MTCs are sporadic, but approximately 20% of MTCs are a result of a germline genetic gain-of-function mutation in the rearranged during transfection (RET) proto-oncogene. Hereditary MTC can be seen in isolation or as part of the multiple endocrine neoplasia (MEN) syndrome type 2A or 2B.

Because calcitonin is mainly produced by C cells of the thyroid gland, the measurement of calcitonin concentrations in blood reflects C cell activity and can therefore be used as a tumor marker for MTC [3]. However, there are many limitations to using calcitonin as a screening method. These limiting factors include problems of cost-benefit, lab methods, false positives and low prevalence of MTC. Cost-benefit is a major concern; one cost-effective analysis has shown that the addition of calcitonin screening to current American Thyroid Association guidelines for the evaluation of thyroid nodules would cost $11,793 per life years saved leading to a subsequent $1.4 billion societal fee [4]. Secondly, measurement of calcitonin levels can be challenging and variable depending on lab methods. Calcitonin is a hormone with molecular heterogeneity as it may exist in both bioactive and immature forms in serum and tumor tissue. This characteristic of calcitonin is one factor that can lead to measurements which vary widely as different assays exploit antiserum that recognize different epitopes of the hormone, leading to variable measurement values [5]. Lastly, false positives may occur frequently in both basal and stimulated measurements of calcitonin [6]. Benign pathologies causing an increase in calcitonin levels include benign hyperplasia of C cells, benign thyroid nodules, differentiated thyroid carcinoma and Hashimoto thyroiditis [7, 8].

Although carcinoembryonic antigen (CEA) has also been proposed as a tumor marker for MTC, it’s trends after surgery and in correlation with calcitonin have not been thoroughly studied in the literature. The limiting factor of low incidence of MTC in the population has led to few studies evaluating CEA in the context of MTC tumor markers, biochemical cure and prognosis, and the ones that exist have shown conflicting results. Machens et al. have studied the implications of preoperative biomarkers including CEA levels on the management of MTC, and concluded that abnormal CEA levels heralds advanced disease, consisting of larger tumors and metastasis [9]. They also propose the addition of biochemical stratification of patients as part of a standardized approach to MTC in order to minimize surgical morbidity [10]. They conclude that further studies are necessary to confirm the effectiveness CEA measurements in risk-stratifying MTC patients. However, in contrast to their findings, Yip et al. discovered that only calcitonin, and not CEA, reflected the extent of disease [11]. They did not find any correlation between preoperative CEA levels and tumor size, lymph node metastasis, or extent of the operation performed.

The purpose of this study is to help clarify the discrepancies in the literature and determine whether abnormal CEA levels may be used as a tumor marker to predict the severity of disease in MTC in regards to size of tumor, stage of tumor, lymph node involvement, distant metastasis, surgical cure, and mortality. As CEA measurements are less costly to the system compared to calcitonin, we hypothesize that it could be a more cost-effective tool in monitoring MTC.

## Methods

### Study design and population

Our study is an analysis of MTC patients who underwent a total thyroidectomy with or without a selective or radical neck dissection at two tertiary surgical centers in Montreal, including the Jewish General Hospital and the Royal Victoria Hospital between the years of 2003–2016. The inclusion criteria included all MTC patients who had pre-operative and post-operative calcitonin and CEA levels, bloodwork which has become part of a routine workup in our institutions. Informed consent was obtained before each surgical procedure that represented standard practice of care.

### Operative approach and histopathological examination

The extent of the primary surgery was at the discretion of the treating physician. Patients underwent either total thyroidectomy or total thyroidectomy with prophylactic central and/or lateral compartment lymphadenectomy. During the operation all thyroid specimens and neck dissections were oriented and marked in the operating room, and then sent for pathological analysis post-operatively. All tissue specimens were embedded in paraffin and subjected to histopathological examination and calcitonin immunohistochemistry. Histological diagnosis, tumor size, depth of invasion, margins, extra-capsular extension, lymphovascular invasion, C-cell hyperplasia and lymph node involvement was then examined by expert thyroid pathologists at our institution. When multiple MTC were present, the largest tumor dimension was considered. Distant metastasis was diagnosed with radiological evidence (ultrasonography, computed tomography, magnetic resonance imaging, positron emission tomography, or any combination thereof) and confirmed by tissue diagnosis when feasible. The American Joint Committee on Cancer 7th edition, TNM staging, was chosen for tumor staging.

### Statistical approach

Various categorical variables were tested using a univariate analysis. In order to study dose effects, continuous variables such as primary tumor size were grouped in increments of 10 mm. Multivariate conditional logistic regression models were then fitted to identify histopathologic variables associated with an abnormal preoperative CEA test result. The level of significance was set at *p* < .05.

## Results

The study included a total of 33 patients (21 females and 12 males) with an average age of 58. The general characteristics of our population were similar to those in other studies; including mean age at surgery = 58, sex ratio = 1 male: 1.75 female, stage *I* = 15.1%, stage II = 27.3%, stage III = 9.1%, and stage IV = 42.4%. All patients underwent surgery, and 54.5% of these patients were biochemically cured. Survival was 93.9% at 10 years. Multivariate analysis showed that age and stage were independent predictive factors of survival (Table 1).

**Table 1:** Multivariate logistic regression analysis - preoperative CEA levels and medullary thyroid cancer progression

	# of patients	Pre-Op CEA (ng/ml)	(95% CI)	*P*-value
Primary tumor size (mm)				
1–9	3	3.36	(1.7-10)	0.01
10–19	7	6.42		
20–29	5	16.54		
30–39	8	168.63		
40+	10	188.73		
Stage				
I	5	2.7	(3.8-130)	0.04
II	11	38.8		
III	3	15.7		
IVa	8	74.7		
IVb	1	128.1		
IVc	5	405.9		
Metastasis				
Locoregional	12	20.55	(−14.4–225.7)	0.08
Distant	6	405.83		
Location of lymph node metastasis				
Central	30	36.1		
Central + Lateral	18	120.1	(19–228)	0.02
Central + Lateral + Contralateral	12	162.31		
Biomedical Cure				
Yes	17	48.3	(8.5–244)	0.03
No	16	174.6		
Mortality				
Yes	2	74.9	(0.0006–0.0013)	< 0.001
No	31	583.7		

### Tumor size

**Fig. 1:**
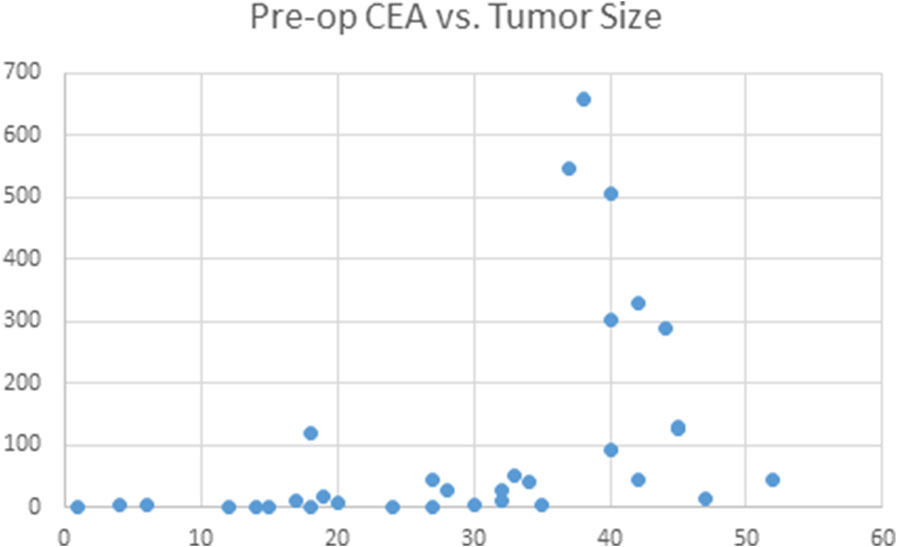
Relationship between pre-operative carcinoembryonic antigen levels and the size of the tumor (mm)

Univariate analysis demonstrates an exponential relationship between pre-operative CEA levels and the size of the tumor as displayed by graph / Table 2. This relationship demonstrates that CEA levels are normal to mildly elevated with small tumor sizes, the pre-operative CEA levels rises significantly once the tumor reaches a size greater than 30 mm (Fig. 1, Table 2).

**Table 2:** Relationship between the size of the tumor and pre-operative carcinoembryonic antigen levels

Size of tumor (mm)	Pre-OP CEA (ng/ml)
1–9	3.36
10–19	6.42
20–29	16.54
30–39	168.63
40+	188.73

### Stage

**Fig. 2:**
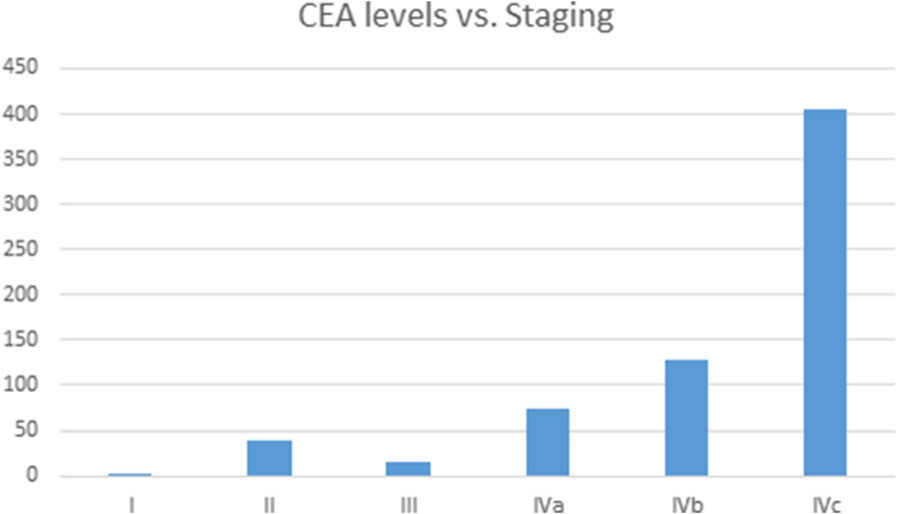
Relationship between pre-operative carcinoembryonic antigen levels and the stage of tumor

A moderate increase in CEA levels is present with stage I, II and III disease, while the CEA levels rise significantly with stage IVC disease, reaching an average of 405.9 ng/ml (Fig. 2, Table 3).

**Table 3:** Relationship between stage of tumor and pre-operative carcinoembryonic antigen levels

Stage	Pre-op CEA (ng/ml)
Stage I	2.7
Stage II	38.8
Stage III	15.7
Stage IVA	74.7
Stage IVB	128.2
Stage IVC	405.9

### Extent of surgery, metastasis and lymph node involvement

Out of our 33 patients who underwent total thyroidectomy, 30 had central compartment lymphadenectomy, 18 had central and ipsilateral neck dissections, while 12 underwent central and bilateral neck dissections (both ipsilateral and contralateral). The average pre-operative CEA for all patients was 94.4 ng/ml, which increased to 103.4 ng/ml for those who had central compartment dissections, 148.3 ng/ml for central and ipsilateral neck dissections, and 162.31 ng/ml for central and bilateral neck dissections. This represents a correlation between pre-operative CEA levels and the extent of the surgical procedure performed.

In total, 18 had metastasis of their disease outside the thyroid gland. The metastasis was divided into locoregional vs. distant metastasis. Of the 18 patients with metastasis, 12 had locoregional and 6 had distant metastasis. These patients had average pre-operative CEA levels of 20.6 ng/ml and 405.8 ng/ml respectively. As with tumor size and staging, the pre-operative CEA levels were seen to be drastically increased in distant metastasis compared to that of locoregional disease (Table 4).

**Table 4:** Relationship between metastatic medullary thyroid cancer (logoregional vs. distant) and pre-operative carcinoembryonic antigen levels

Metastasis	Pre-Op CEA (ng/ml)
Locoregional	20.55
Distant	405.83

Presence of disease in the contralateral neck compartment was also associated with higher CEA level measurements. While presence of positive lymph nodes only in the central neck compartments were seen with an average pre-operative CEA of 103.39 ng/ml, lateral neck and contralateral neck lymph node findings were associated with a much higher CEA level at 120.1 ng/ml and 162.31 ng/ml respectively (Table 5).

**Table 5:** Relationship between location of lymph node metastasis and pre-operative carcinoembryonic antigen levels

Location of LN	Pre-Op CEA (ng/ml)
Central only	318.6
Central and lateral	100.3
Lateral only	91.1

**Fig. 3:**
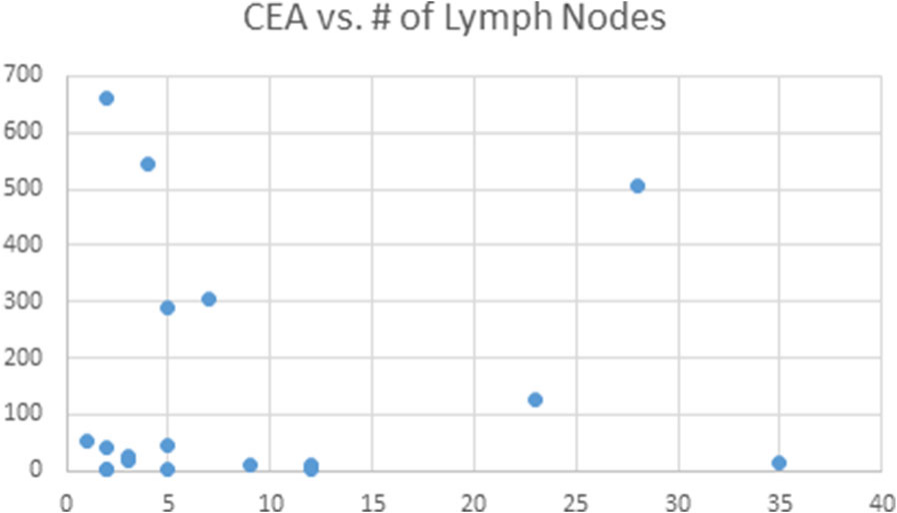
Relationship between pre-operative carcinoembryonic antigen levels and the number of metastatic lymph nodes

Surprisingly, no correlation with the number of metastatic cervical lymph node involvement was seen. Both minimal lymph node involvement and extensive lymph node metastasis had the potential to correlate with high CEA levels (Fig. 3).

### Biochemical cure

Chances of biochemical cure, defined as normal basal post-operative serum CEA and calcitonin levels, were greatly diminished in patients who had higher pre-operative CEA levels (Tables 6).

**Table 6:** Relationship between biochemical cure and pre-operative carcinoembryonic antigen levels

Biochemical Cure	Pre-Op CEA (ng/ml)
Yes	48.3
No	174.6

### Mortality

In total, two patients succumbed from their disease. The pre-operative CEA levels of these patients were 659.9 and 507.4, representing the two patients in our population with the highest CEA levels and demonstrating that significantly increased CEA levels can be associated with mortality.

### Calcitonin

**Fig. 4:**
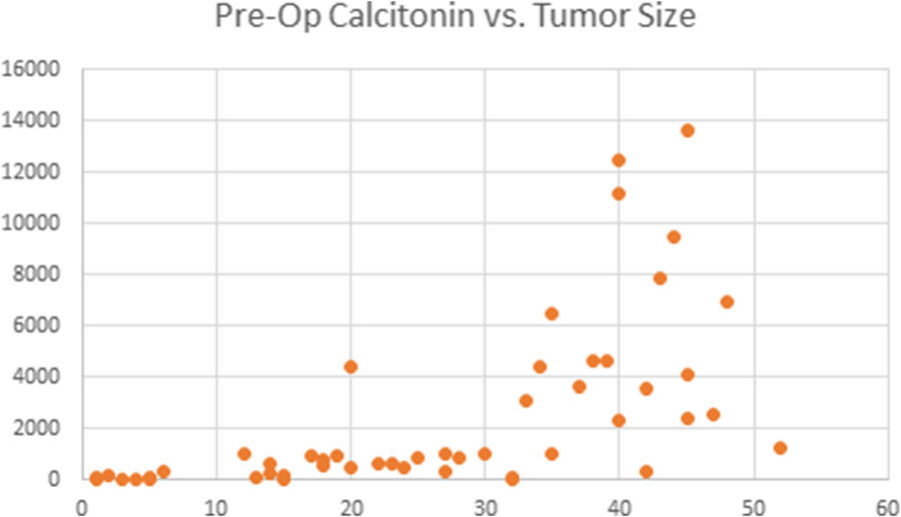
Relationship between pre-operative calcitonin levels and the size of the tumor (mm)

Although not the focus of this study, pre-operative calcitonin levels were also compared to extent of medullary thyroid cancer progression in tumor size. Univariate analysis demonstrated a more linear relationship between pre-operative calcitonin levels and the size of the tumor compared to that of CEA as displayed by Fig. 4.

## Discussion

MTC, a relatively rare type of thyroid malignancy, is associated with a poor survival when compared to other common thyroid cancers. Our cohort had a 93.9% 10-year survival rate which is similar to previous studies reporting survival rates ranging from 69 to 89% [12–14]. As with other studies, our current analysis is in agreement with previously identified clinical prognostic factors for MTC including age (> 45 years), tumor stage, presence of lymph node and distant metastasis.

MTC is known to produce many tumor markers, including calcitonin, CEA, and chromogranin A. These markers can be easily detected with blood levels and immunohistological stains. Serum calcitonin is known to be a highly sensitive tumor biomarker, shown to be 100% predictive of MTC when basal levels are > 100 pg/ml or when stimulated levels with pentagastrin increase to > 1000 pg/ml [15]. Although these levels have been proven for calcitonin, a similar measure has not yet been proposed in the literature for CEA levels. CEA is a glycoprotein that was first detected by Gold et al. in 1965 in relation to colon cancer [16]. It is most commonly known as a tumor marker for gastrointestinal malignancies, frequently used to monitor tumor recurrence. In patients presenting with high CEA levels, tumors originating from the gastrointestinal tract should always be excluded using endoscopy, colonoscopy, and CT. CEA levels may also increase in benign disease, including inflammatory bowel disease and liver cirrhosis. Despite this, many cases have been noted in the literature in which elevated CEA levels with no other clinical findings may be the first and only finding of MTC [17].

It has been shown in the past that production of various tumor markers may differ between patients and that tumors with a high production of CEA alongside a low expression of calcitonin may be more aggressive [18]. It was postulated that this finding may reflect a degree of maturation block of tumor cells in patients with aggressive disease. However, this “flip-flop phenomenon” was not seen in our study, as the patients with the highest tumor burden (represented by presence of distant metastasis and mortality) also had the highest calcitonin levels in our cohort. While calcitonin is thought to have a linear relationship with disease progression, our analysis discovered that abnormal CEA levels were exponentially correlated with advanced disease. This suggests that CEA can be a better predictor of advanced disease and mortality, in agreement with previous studies suggesting CEA may be a sensitive marker for aggressive MTC [19]. CEA was consistently elevated in all cases with metastasis, whereas calcitonin may be normal or moderate with metastatic presence. This may suggest that using CEA levels in correlation with calcitonin may detect disease metastasis or recurrence earlier than calcitonin alone. Our results also demonstrate that CEA is a better detector of lateral and contralateral lymph node involvement, which may help in guiding surgical approaches.

Interestingly, the only variable in which we saw no correlation with disease severity was the number of metastatic cervical lymph nodes. Although the presence and location of metastasis was significant for advanced disease, the specific number of pathologically proven lymph nodes had no correlation with CEA levels. Previous research has proposed that the number of lymph nodes harboring metastasis can be used as an independent prognostic factor in differentiated thyroid cancer [20]. However, in keeping with our findings, these papers also concluded that although biomarker levels correlate closely with tumor mass, they often don’t have as strong of a predictive value in the number of lymph node metastasis. Some theories include that while the tumor mass itself has excellent vascular supply and drainage, lymph node metastasis may exist in area of reduced perfusion thus correlating more poorly with pre-operative CEA and calcitonin levels. It is also contemplated that lymph node metastasis may have acquired additional somatic mutations rendering these cells less differentiated than those of the primary tumor and thus leading to reduced correlation with serum levels.

Although often overlooked, there exists a significant importance on cost-comparison when considering additional screening tests. At our institutions, one serum calcitonin test costs approximately $30, compared to $4 for a serum CEA level, which theoretically could cut screening costs by 87%. Furthermore, it is also important to note that at many institutions CEA is analysed rapidly (comparable to the way blood glucose levels are analysed and resulted) whereas calcitonin levels are requested much less frequently requiring outsourcing and delays in receiving laboratory values.

This population-based study evaluating CEA tumor marker levels on MTC progression has several potential limitations. First, MTC is quite rare compared to other differentiated thyroid cancers, leading to small study population. Second, MTC occurs in both hereditary and sporadic forms. As sporadic forms tend to be more aggressive, this may confound our findings as our population consisted of 94% sporadic form of MTC. Lastly, as our institution is that of a tertiary center, the referral pattern may also lean towards a biased increase in patients with aggressive disease. Despite these limitations, the findings of our project have guided some new clinical applications in our practice. Currently, all patients with diagnosed MTC receive pre-operative and post-operative CEA levels. In instances of high pre-operative CEA levels, attention is given to counseling the patient on the invasiveness of the tumor and a discussion can take place for more aggressive surgical therapy. Similarly, if post-operative CEA levels are surprisingly high, closer surveillance for the patient may be considered.

## Conclusion

In this study, both pre-operative calcitonin and CEA levels were significantly correlated with the extent of disease in MTC. While calcitonin has a linear relationship with disease progression, abnormal CEA levels were also correlated with advanced disease suggesting that it also may be a predictor of tumor size, central lymph node metastasis, and mortality. CEA levels greater than 271 ng/ml are significant for advanced tumor size, advanced tumor staging, metastasis to the contralateral neck compartment, and decreased chance of biochemical cure. CEA levels greater than 500 ng/ml are greatly associated with patient mortality.

## Abbreviations

CEA: Carcinoembryonic antigen
CT: Omputed tomography
MEN: Multiple endocrine neoplasia
Mtc: Medullary thyroid cancer
RET: Rearranged during transfection
